# Hierarchically Porous Coatings for Cellulose Fibers by Core–Shell Particle Templating

**DOI:** 10.1002/marc.70293

**Published:** 2026-04-27

**Authors:** Regina Leiner, Derya Kurt, Sebastian Heinz, Volker Presser, Bizan N. Balzer, Markus Gallei

**Affiliations:** ^1^ Polymer Chemistry Saarland University Saarbrücken Germany; ^2^ INM – Institute for New Materials Saarland University Saarbrücken Germany; ^3^ Department of Materials Science and Engineering Saarland University Saarbrücken Germany; ^4^ Saarene, Saarland Center for Energy Materials and Sustainability Saarland University Saarbrücken Germany; ^5^ Institute of Physical Chemistry University of Freiburg Freiburg Germany; ^6^ Freiburg Materials Research Center (FMF) University of Freiburg Freiburg Germany

**Keywords:** cellulose, core–shell, filter, porous coatings

## Abstract

Porous and functional cellulose‐based materials play a key role in the field of novel sensors and membrane technologies, yet their full potential remains unexplored. This article elaborates on a procedure for creating porous coatings with reactive chemical groups on their surfaces by covering a cellulose membrane with hybrid core–shell particles. The silica cores of these particles, synthesized via the Stöber procedure, could easily be etched with hydrofluoric acid. The cross‐linked polymer shell of the particles was synthesized via emulsion polymerization. These particles were analyzed via dynamic light scattering and transmission electron microscopy. After coating the cellulose and an etching process, the former core particles formed pores within the matrix of the shell polymer, as observed via scanning electron microscopy and atomic force microscopy. The coated area also featured chemical functionalities via appropriate polymers used in the shell of the particles, enabling further cellulose modification. In particular, hydroxy groups were incorporated into a copolymer containing 2‐hydroxyethyl methacrylate, and epoxy groups were incorporated using glycidyl methacrylate. These functionalities can be combined to yield a wide range of specific properties. For this reason, this work paves the way for advanced smart and stimuli‐responsive porous filtration systems, paper‐based sensors, and adsorbers.

## Graphical abstract

Hybrid and functional core–shell particles comprising a silica core and a polymer shell were synthesized and used to develop a coating and etching process applied to a cellulose substrate. This resulted in highly porous, functional coatings on a cellulose membrane for further application in cellulose‐based adsorbers and advanced membrane technology.

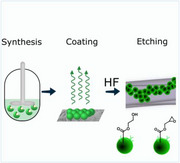

## 1 Introduction

Cellulose is an abundant, renewable, and non‐toxic biopolymer, and therefore a valuable and environmentally friendly resource [1, 2]. Due to the presence of large and accessible feedstocks of cellulose fibers, along with their properties, such as high strength and stiffness at low weight, numerous applications of this class of bio‐sourced materials are known today. For example, cellulose‐based materials can exhibit adjustable morphologies while maintaining high porosity. This results in beneficial transport and separation properties, making cellulose ideal for films, filter papers, and membranes [3, 4]. The positive effect of the high porosity can also find its application in the form of hybrid systems using the interplay of porous organic and inorganic materials. Hierarchically structured hybrid systems are widely used as catalysts, for energy storage, or for selective separations [5, 6, 7]. Nowadays, so‐called functional and hybrid cellulose papers are used for technologies such as printed electronics, capacitors, and sensors [8, 9, 10, 11]. Therefore, functional papers that allow direct and technologically simple control over the wettability, swelling properties, or functionality through surface modification are suitable as platforms for microfluidic or sensor applications [12, 13]. Methods to adapt surface functionality could be established through a multitude of materials with predefined properties, either directly through chemical reactions or as a coating material. For example, polymer latex particles could be used to create a film covering the cellulose substrate [14]. Depending on the functionalities of the used polymer, different surface properties could be achieved [15]. Equivalently, stimuli‐responsive properties could be realized by using appropriate functionalities and polymers in numerous surface modification techniques.

However, a full understanding of the modification of cellulose fibers with polymers is not yet present today. These findings concern not only the influence of the grafted architecture but also pore formation and the number of confined functionalities. The latter are based on pore‐building segments and complex polymer architectures at the cellulose fiber surface. Understanding the polymer domain orientation and self‐assembly processes of polymers near cellulose fibers, as well as the resulting paper confinement, would significantly expand the application potential of paper‐based microfluidic devices for novel sensors, membrane technologies, and smart sorption technologies. A research field that enables the formation of structure in the bulk state or for the preparation of well‐defined pores is the self‐assembly of block copolymers [16, 17]. Another route to obtain porosity is based on inverse opals [18, 19]. In these cases, particles within a cross‐linked matrix are established. These particles could be aligned in a close‐packed arrangement and serve as a template, allowing the system to form a porous structure after the particles are removed using suitable etching procedures [20, 21]. Core–shell particles (CSPs) are promising candidates for the formation of such inverse opal structures. The core particles correspond to the template for the later pores, whereas the shell of the particles forms the later matrix of the porous system [22, 23, 24].

In the present work, additional porosity and surface modification of cellulose paper sheets were achieved using CSPs. For this reason, CSPs containing silica cores and a copolymer shell were synthesized via emulsion polymerization and used as coatings onto cellulose fibers. Recently, we showed that CSPs can adhere to cellulose fibers, revealing structural colors arising from the high degree of particle order at the cellulose fiber surface [25, 26]. However, the preparation of inverse opal structures on the surface of highly porous cellulose fibers and fiber networks thereof has not been reported yet. An appropriate method yielding such a hierarchical pore structure would enable precise solvent flux control in particle‐based materials, given the different pore sizes within the material. As a conclusion, the herein described preparation strategy would enable the tailored design of paper‐based sensors with intelligent, stimuli‐responsive functionalities, which will be part of future studies. However, the preparation of inverse opal structures on the surface of cellulose fibers has not been reported yet. This would enable the tailored design of cellulose‐based substrates with intelligent functionalities, such as stimuli‐responsive moieties and the formation of hierarchically ordered pore structures. In this study, two separate approaches were used, in which the shell material was once a copolymer of ethyl acrylate and 2‐hydroxyethyl methacrylate (P(EA‐*co*‐HEMA)). Second, particles with an outer shell of a copolymer using *n*‐butyl acrylate and glycidyl methacrylate, P(*n*BuA‐*co*‐GlyMA), were synthesized. Both particle batches contained functional groups, allowing subsequent post‐treatments to convert them into other functionalities. Cross‐linking the matrix for enhanced stability and etching the silica particles constitute a strategy for adding porous structures to a polymer‐coated cellulose substrate. The resulting coated surface was analyzed for morphology using scanning electron microscopy and atomic force microscopy. Water contact angle measurements were performed to assess the coating's effect on the hydrophilicity of the cellulose filter sheet.

## 2 Experimental Section

### 2.1 Materials

2‑hydroxyethyl methacrylate (HEMA, 97%), benzophenone (99%), and ammonia (25%) were purchased from Fisher Scientific. Methyl methacrylate (MMA, 99%), ethyl acrylate (EA, 99.5%), glycidyl methacrylate (GlyMA, ≥97%), butanediol diacrylate (BDDA, technical grade), potassium hydroxide flakes (KOH, 90% reagent grade), sodium disulfite (NaDS, analysis grade), sodium persulfate (NaPS, ≥98%), sodium dodecyl sulfate (SDS, ≥98.5%), tetraethyl orthosilane (TEOS, ≥99%), vinyl methacrylate (VMA, 98%), and 2‐hydroxy‐2‐methylpropiophenone (HMPP, 97%) were purchased from Sigma–Aldrich. Allyl methacrylate (ALMA, 98%) and *n*‐butyl acrylate (*n*BuA) were purchased from TCI. Dowfax2A1 was purchased from EZkem. Ethanol (99.5%, denaturated with 1% MEK) was purchased from VWR Chemicals. 3‑(Methacryloxy)propyl trimethoxysilane (MEMO, 98%) was purchased from abcr. Irgacure 184 was obtained from BASF. Crelan UI was purchased from Covestro. Hydrofluoric acid (HF, 40%) was purchased from Merck. Munktell, cellulose‐based filter discs from Ahlstrom Munksjö (grade 3hw, 65 g cm^−3^, 3.303.070), were used as substrate.

Before emulsion polymerization, stabilizing agents were removed from the monomers by passing them through a basic alumina column (50–200 µm, Acros Organics). Exceptions were made for HEMA, where neutral aluminum oxide (40–300 µm, Thermo Scientific) was used. For GlyMA, the column was treated with THF before using the neutral alumina column.

### 2.2 Instrumentation

Dynamic light scattering (DLS) measurements were once performed using a Zetasizer Nano ZS90 (Malvern) equipped with a 4 mW, 633 nm HeNe laser at 25°C. Size was measured at an angle of 90° with a five‐fold determination of 15 runs. Automatic data acquisition in 300 size classes and peak determination was done using Zetasizer Nano software. The intensities were normalized to the highest signal in every average measurement. The results shown for the GlyMA‐containing particles were measured on a Kalliope Litesizer DLS500 (Anton Paar) at an angle of 90° over 6 measurement runs after filtering the sample through regenerated cellulose with a pore size of 0.20 µm. For transmission electron microscopy (TEM), the diluted polymer dispersions were drop‐cast on a carbon‐coated copper grid. The dispersion medium evaporated at ambient conditions for at least 12 h. TEM measurements were conducted using a JEOL JEM‐2100 LaB_6_ electron microscope (JEOL) at a nominal acceleration voltage of 200 kV with a Gatan Orius SC100 CCD camera in bright‐field mode. Particle sizes were analyzed using ImageJ. Scanning electron microscopy (SEM) was carried out on a Zeiss Gemini500 Sigma VP device using SmartSEM Version 6.07, with accelerating voltages between 2 and 4 kV. Before, the particle samples were mounted on an aluminum stub using adhesive carbon tape and coated with 6 nm platinum using an Automatic Turbo Coater PLASMATOOL 125 SIN 2020_131 from Ingenieurbüro Peter Liebscher. Differential scanning calorimetry (DSC) was performed with a Netzsch 214 Polyma with a heating rate of 10 K min^−1^ and a sample mass of 5.0 mg. The glass transition temperature was determined using Netzsch Proteus software. Water contact angle measurements were performed by adding 10 µL of distilled water per droplet, using a Hamilton syringe in a syringe pump by kdScientific and a custom xyz positioning table. Photographs were collected using a Nikon D54000 and digiCamControl 2.1.2.0, opendrop 3.3.1 was used for evaluation. For atomic force microscopy (AFM)‐based imaging, the Cypher ES (Asylum Research, an Oxford Instruments Company) was used in intermittent‐contact mode (AC mode, Piezo driven) at ca. 22°C in air with AC240TS cantilevers (Olympus; spring constant: approx. 2 N m^−1^, resonance frequency in air: approx. 70 kHz, tip radius: approx. 5 nm). Imaging was performed with 512∙512 pixels, a scan rate of 2.44 Hz, and a scan angle of 90°, with the fast scan axis perpendicular to the long cantilever axis. The samples were glued onto mounting pucks using carbon tape. For evaluation, the images were processed via the Gwyddion Free SPM analysis software version 2.60 [27], applying the following operations on the z sensor retrace data for topography information: the color scale gwyddion.net was used for the presentation of the images. When required, the data were levelled (plane subtraction), scar artifacts were removed, and line‐by‐line correction was performed using row alignment (median of differences). The minimum data values in each image were shifted to zero (fix zero function). The images were presented using non‐linear color scales to better display details. Line profiles were taken with a width of 1 pixel, respectively. Diameter values for the CSPs and pores have usually been taken from 10 values, determined at different spots, using the full width at half maximum (FWHM) of the local topography maxima corresponding to the CSPs or pore rims, respectively. The error values correspond to the standard deviation (1 σ). Thermogravimetric analysis (TGA) was performed with a Netzsch TG 209 F1 Libra system with a heating rate of 10 K min^−1^ and a sample mass of 15.0 mg. The onset of thermal degradation was determined using Netzsch Proteus software.

### 2.3 Synthesis and Functionalization of Silica Core Particles

Following the procedure for the stepwise growth of silica particles using TEOS in ammonia and ethanol, described by van Blaaderen et al. [28], silica particle dispersions with a final solid content of 2.5 mass% were prepared. To use these particles as cores in emulsion polymerization, they were functionalized with MEMO, as described in earlier publications, and the solvent was exchanged with deionized water [22, 23]. The functionalized particles were obtained with a solid content of 9.77±0.04 mass% (7.90±0.21 mass%).

### 2.4 Synthesis of Interlayer and Shell

Using emulsion polymerization, 112.6 mL (55.7 mL) of the functionalized silica particle dispersion was filled in a 250 mL double‐wall reactor equipped with a reflux condenser and a stirrer under a nitrogen atmosphere at 85°C. While stirring at 300 rpm, the emulsion polymerization was initiated by adding 0.14 g (0.06 g) NaDS and 0.12 g (0.05 g) NaPS. After a reaction time of 15 min, the monomer emulsion for the interlayer, ME1, containing 0.99 g (0.38 g) MMA, 0.20 g (0.05 g) ALMA, 0.02 g (0.01 g) Dowfax2A1, 0.01 g (0.005 g) SDS, 5.53 g (2.22 g) deionized water, and 0.55 g (0.22 g) KOH was continuously added with a constant flow rate of 0.25 mL min^−1^ using a rotary piston pump. 15 min after the complete addition of ME1, the emulsion polymerization was reinitiated by adding 0.12 g (0.05 g) NaPS and was continuously stirred for another 10 min. A second monomer emulsion, ME2, for the outer shell was prepared, containing 0.04 g (0.02 g) SDS, 0.03 g (0.03 g) Dowfax2A1, 0.41 g (0.17 g) KOH, and 11.5 g (4.6 g) deionized water. As monomers, 8.91 g EA and 0.99 g HEMA (3.55 g *n*BuA and 0.39 g GlyMA) were emulsified in ME2; the latter was continuously added to the reinitiated particles at a flow rate of 0.25 mL min^−1^ using a rotary piston pump. After complete addition of the ME2, the reaction mixture was held at constant temperature and stirred for an additional hour.

### 2.5 Coating Preparation

The particle emulsion was mixed with cross‐linking agents, where Crelan UI (3 mass%) was used for particles with HEMA in the outer shell, and the mixture was drop‐cast onto cellulose linters filter paper using a 3 × 3 cm 3D‐printed template. The paper sheets were cross‐linked for 10 min at 190°C. Concerning the CSPs with GlyMA, 0.418 g of the particle emulsion was mixed with two droplets of VMA and HMPP, each. After drop‐casting, these filter papers were cross‐linked in a UV‐cube (UVA Cube 2000 from Hoenle, equipped with a UVAPRINT 100–200 HPZ ET lamp with a range of 200–450 nm) at 1000 W for 20 min.

### 2.6 Etching

The silica cores were etched by immersing the coated cellulose sheets in a 2 mass% HF solution for 3 days, unless otherwise stated. After this process, the samples were washed several times with deionized water.

## 3 Results and Discussion

### 3.1 Synthesis of Hybrid Core–Shell Particles

**SCHEME 1:**
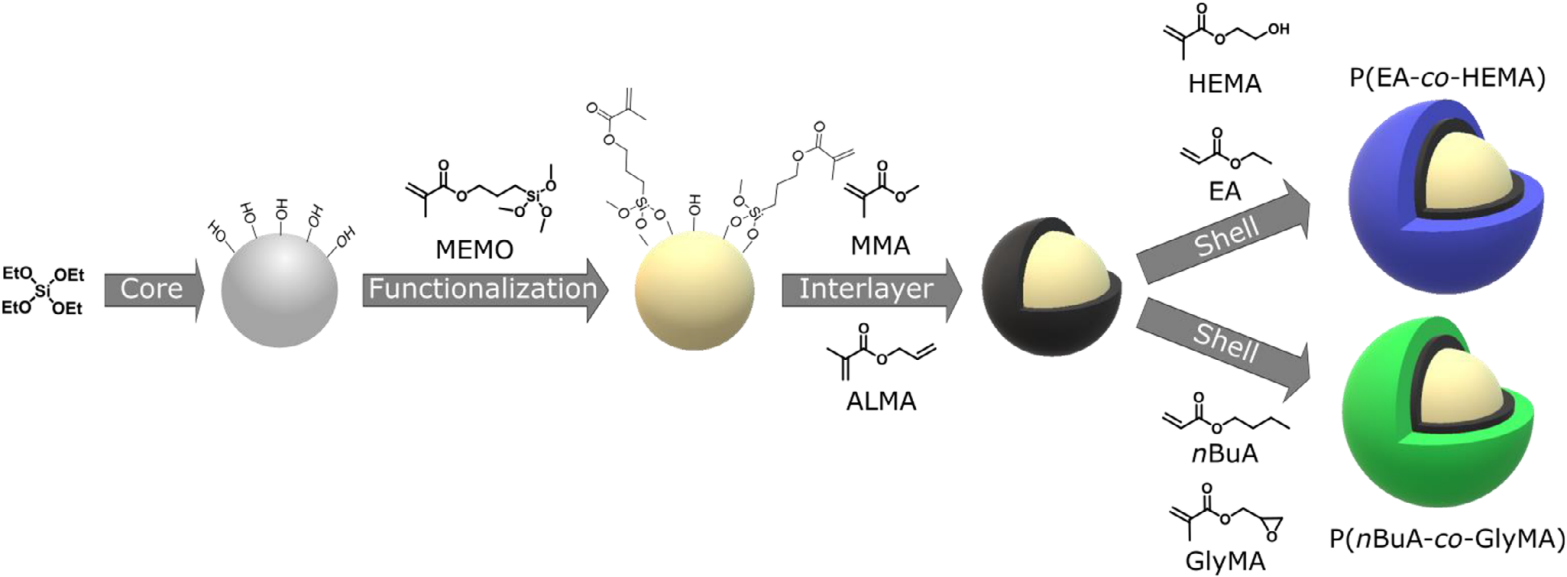
Synthesis scheme of the CSPs. From left to right: TEOS was used to synthesize the silica particles, which were further functionalized with MEMO. Used as cores, the particles received first a polymer layer of MMA and ALMA (black particles), and either an outer layer of EA and HEMA (blue particles), or *n*BuA, and GlyMA (green particles), respectively.

For obtaining hybrid CSPs, monodisperse silica spheres were used as core material and synthesized following a stepwise Stöber procedure, as described in the Experimental Section. After functionalization with MEMO, the silica particles contained methacrylate groups on the surface, making them attractive for further use in emulsion polymerization to add a polymer layer. This way, an immobilized and cross‐linkable interlayer of a copolymer containing ALMA and MMA was added. In the next polymerization step, a soft shell was created, as displayed schematically in Scheme 1. In this study, two different functionalities were implemented separately in the outer shell, using HEMA or GlyMA. As the therewith produced polymers contain hydroxyl or epoxide groups, respectively, they enable further chemical modification, for example, the introduction of responsive functionalities.

**FIGURE 1:**
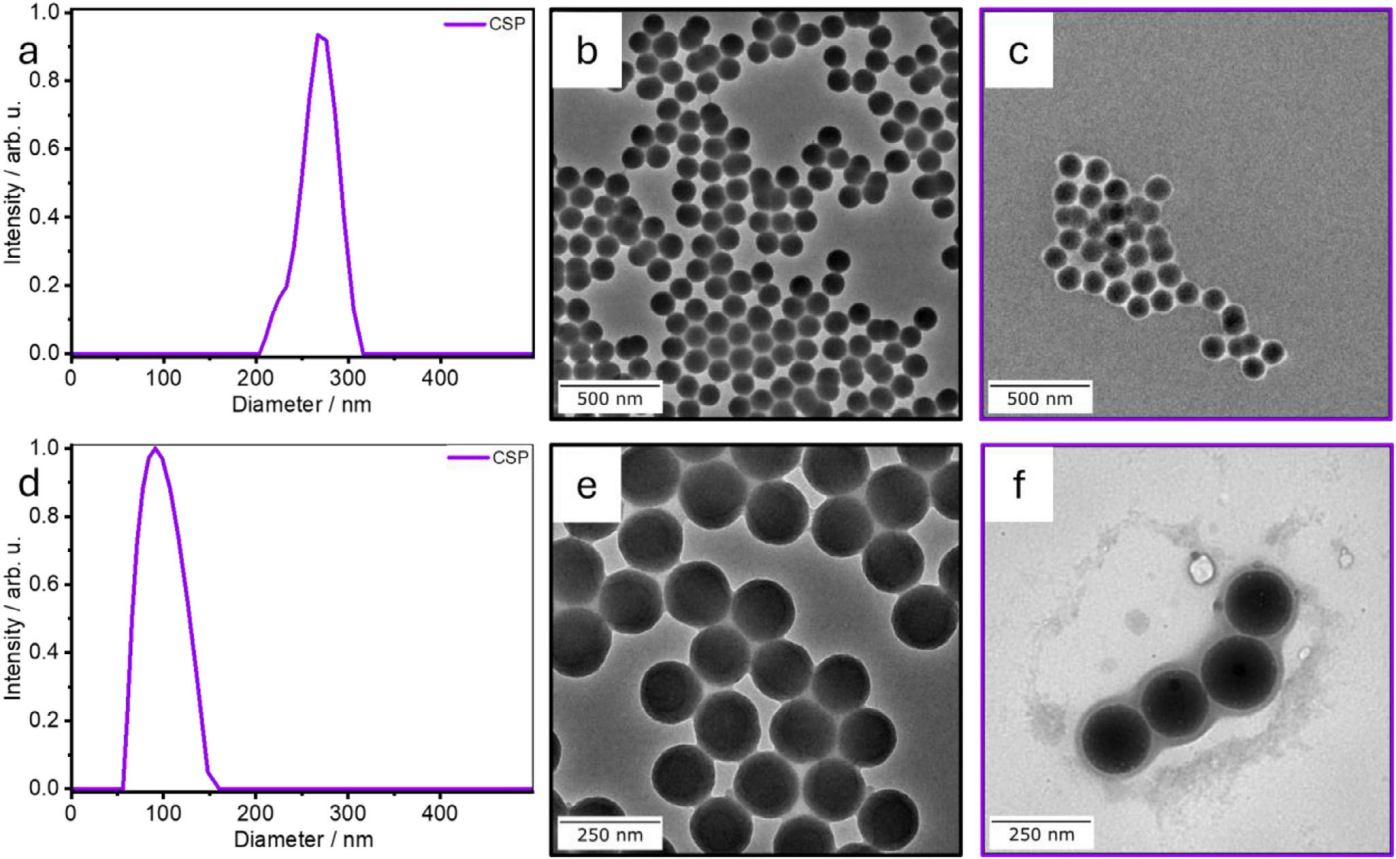
Analysis of the synthesized CSPs. (a) DLS analysis, (b) transmission electron micrograph of the core particles, and (c) transmission electron micrograph of the CSPs containing P(EA‐*co*‐HEMA). (d) DLS analysis, (e) transmission electron micrograph of the core particles, and (f) transmission electron micrograph of the CSPs containing P(*n*BuA‐*co*‐GlyMA).

The first type of CSPs contained a copolymer of 90 mass% EA and 10 mass% HEMA in the outer shell. These CSPs are also referred to as P(EA‐*co*‐HEMA) particles. This particle composition was chosen in accordance with previous studies that elucidated attractive interactions with cellulose, resulting in stronger adhesion properties than those of CSPs without hydroxy groups [25, 29]. The shell growth of the CSPs was investigated, as shown in Figure 1a–c and in Figure S1a, using DLS of samples taken during and after the emulsion polymerization, as well as via TEM. According to these analyses, the core–shell particles reached a size of 265±21 nm, corresponding to a shell content of 56 vol% in the swollen state in the DLS measurements, and 135±8 nm (23 vol% shell) in the dried state when using TEM.

To provide addressability of other chemical functionalities after the processing step, CSPs with an outer shell containing 90 mass% *n*BuA and 10 mass% GlyMA were synthesized and are further described as P(*n*BuA‐*co*‐GlyMA) particles. After polymerizing an outer shell, the averaged results of the DLS measurements showed particles with a hydrodynamic diameter of 98 nm with a polydispersity of 4%, revealing a monodisperse distribution (Figure 1d). The corresponding DLS measurements of the core particles, as well as the particles covered with the interlayer, are shown in Figure S1. For comparison in the dried state, these CSPs were measured via TEM, indicating a size of 188±13 nm, corresponding to 51 vol% shell compared to the pure core particles with a size of 148±8 nm, as depicted in Figure 1e,f.

In summary, CSPs with a silica core, a cross‐linked copolymer interlayer, and a copolymer shell were successfully synthesized using the Stöber process, followed by emulsion polymerization. The particles could be distinguished by their size and shell content, 135 nm and 56 vol% shell for the HEMA‐containing particles, against 188 nm and 51 vol% for the GlyMA‐containing particles. When applied as a coating on a substrate, a higher shell volume resulted in greater spacing between the core particles, thereby affecting the final pore spacing and order. Within this first study, CSPs with similar shell content were synthesized, which will not lead to significant differences in the later pore distances. Further, the CSPs possess functional groups on their surfaces, namely hydroxy groups stemming from HEMA or epoxide groups from GlyMA. The Preussmann test was used to prove the presence of epoxide groups by adding 4‐(4‐nitrobenzyl)pyridine, which caused a color change from white to purple (Figure S2) [30, 31, 32].

### 3.2 Porous Coatings onto Cellulose Linters: CSPs using P(EA‐*co*‐HEMA)

To use the synthesized particles in a particle‐based coating procedure for cellulose fibers, first, the CSP emulsion with an outer shell of P(EA‑*co*‑HEMA) was drop‐cast onto a cellulose filter paper, forming a thin layer of particles on the cellulose fibers. For this reason, the particle emulsion with a solid content of 13.2±0.1 mass% was mixed with approximately 3 mass% of a thermal cross‐linker before coating the cellulose filter paper. The thermal cross‐linker was used to stabilize the shell material, thereby forming a rigid network around the silica cores, as it had already been used to cross‐link HEMA‐containing CSPs in opal films [31, 33, 34]. To prevent the emulsion droplet from spreading across a large area on the filter paper due to the hydrophilicity of cellulose and its interactions with the water in the particle emulsion, a 3D‐printed template was used to apply pressure along the borders of a 3×3 cm^2^ area. Another method for applying the CSPs to the cellulose substrate involved several filtration cycles until the desired number of particles was completely retained within the filter paper. To further initiate the cross‐linking reaction between the particles, the coated films were heated to 190°C for 10 min.

**FIGURE 2:**
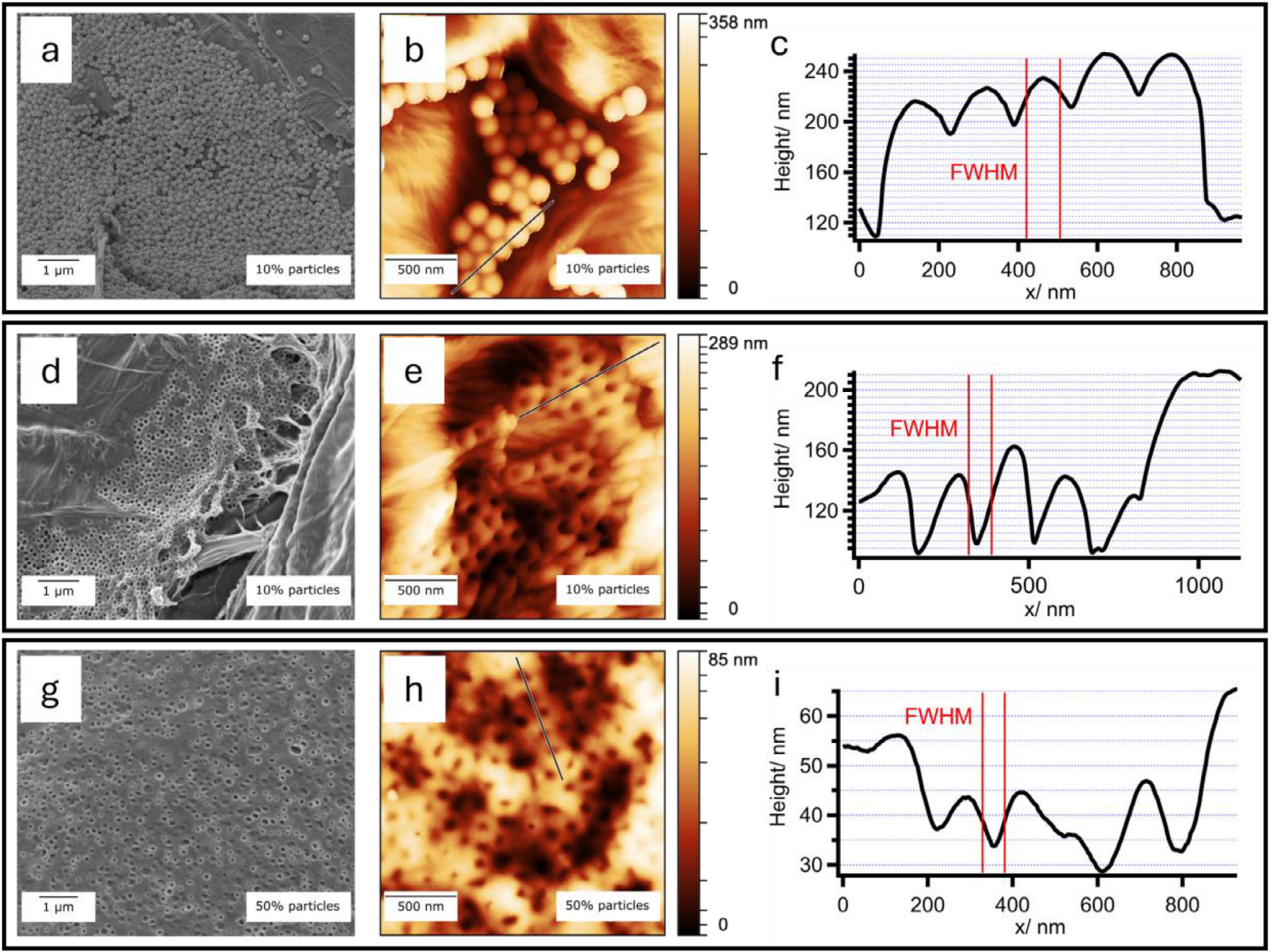
Analysis of the cellulose surface, coated with CSPs with an outer shell of P(EA‐*co*‐HEMA). From left to right, the images show scanning electron micrographs (a,d,g) and AFM scans (b,e,h), along with representative line profiles (c,f,i). (a–f), 10 mass% coating of the CSPs applied, (a–c) before and (d–f) after etching the silica cores. (g–i), 50 mass% coating of CSPs onto the cellulose after etching.

After etching the samples with HF, the former shell polymer formed a continuous coating with pores at the former locations of the silica particles. The previous cross‐linking in the coating was essential to prevent the pores from collapsing. The difference between the intact particles before the etching process and the later porous coating is visible in the scanning electron micrographs, Figure 2a,d. Applying a coating of 10 mass% CSPs resulted in an incomplete coating of the filter paper, with the particles primarily arranged between the cellulose fibers within the substrate. To obtain a uniform coating covering the entire surface of the paper sheet rather than local areas of single fibers, other porous coatings were prepared by adding higher CSP concentrations (20 mass%, 36 mass%, and 50 mass%) relative to the cellulose filter paper. The corresponding scanning electron micrographs of these samples after etching the silica cores are depicted in Figure 2g and Figure S3. Applying 36 mass% of the particles resulted in a uniform, complete coating of the cellulose fibers. This coating was applied across several filtering cycles of the CSP emulsion through cellulose paper. This way, the particles could be found not only on the surface, but also throughout the cellulose film. Increasing the particle mass to 50 % via drop‐casting resulted in a thick layer of particles on the cellulose fibers. In this case, the particles showed greater disorder than in coatings containing 10 mass%.

The limited areas of coated surface at 10 mass% of particles, and the increasing disorder in the coating at 50 mass%, were also visible after the etching procedure. While the pores were arranged in a hexagonal structure (Figure 2e), the order of the pores in the thicker coating decreased (Figure 2h). Also, a less porous structure was obtained using 36 and 50 mass% of particles for the coatings, as the HF less efficiently reached the silica cores within the cross‐section of the film, resulting in still‐present silica particles.

This behavior was also observed when the particles were processed into inverse opal films, as shown in Figure S4. An additional reason for the less porous structure could be a possible collapse of the porous structure, possibly due to inefficient cross‐linking of the matrix. The silica particles were etched successfully in these cases, but the matrix collapsed, and the pores closed.

To improve porosity while maintaining the ordered arrangement of the particles on the cellulose substrate, a coating containing 20 mass% of particles was applied. This porous coating provided both homogeneous fiber coating and pores over the entire surface. To conclude, the drop‐casting method using a coating of 10–20 mass% P(EA‐*co*‐HEMA) CSPs in the outer shell yielded the most homogeneous porous coating after removing the silica cores.

The coated films made with 10% and 50% P(EA‐*co*‐HEMA)‐CSPs were analyzed via atomic force microscopy (AFM) to illustrate their topographies and line profiles. As shown in Figure 2, the AFM images depict the same surface topography as obtained via SEM. The line profiles (Figure 2c,f,i) change at the position of a particle from a local maximum to a local minimum after the silica core particles were etched. This could be deducted from the full width at half maximum (FWHM) of the local maxima as a measure of the CSP and pore diameters, respectively. For the 10 mass% coating, a CSP diameter of 98±14 nm was obtained, based on *n* = 10 values (Figure 2c). The corresponding pore diameters after etching amount to 55±20 nm (*n* = 10 values) (Figure 2f). The AFM cantilever tip influences the obtained topographies [35], shifting the CSP diameters to larger values and the pore diameters to smaller values, compared to their real values. Thus, the obtained pore diameters correspond to little more than half of the CSP diameter. That appears reasonable, as only the matrix of the CSPs is expected to withstand the etching process, whereas the core size determines the final pore size. Similar results were obtained for 50 mass% coating of CSPs, where CSP diameters of 100±31 nm (*n* = 10 values) transformed to pores with a diameter of 56±8 nm (*n*  =  10 values) after etching (Figure 2i; Figure S3b).

Water contact angle measurements of the coated and non‐coated samples, before and after the etching process, demonstrated the influence of the substrates’ surface properties. For comparison, the contact angle of a pure filter paper sheet was measured, where the water droplet was immediately absorbed. Adding a coating of CSPs with the shell P(EA‐*co*‐HEMA) resulted in contact angles of 79±1° (10 mass% CSPs) and 88±3° (20 mass% CSPs), and the water droplet remained on the surface. This increased hydrophobicity indicated successful coating of the cellulose substrate. After etching the cores, the contact angles increased even further, to 117±8° and 95±4°, respectively, due to the increasing porosity and the resulting rough surface.

### 3.3 Porous Coatings onto Cellulose Linters: CSPs using P(*n*BuA‐*co*‐GlyMA)

**FIGURE 3:**
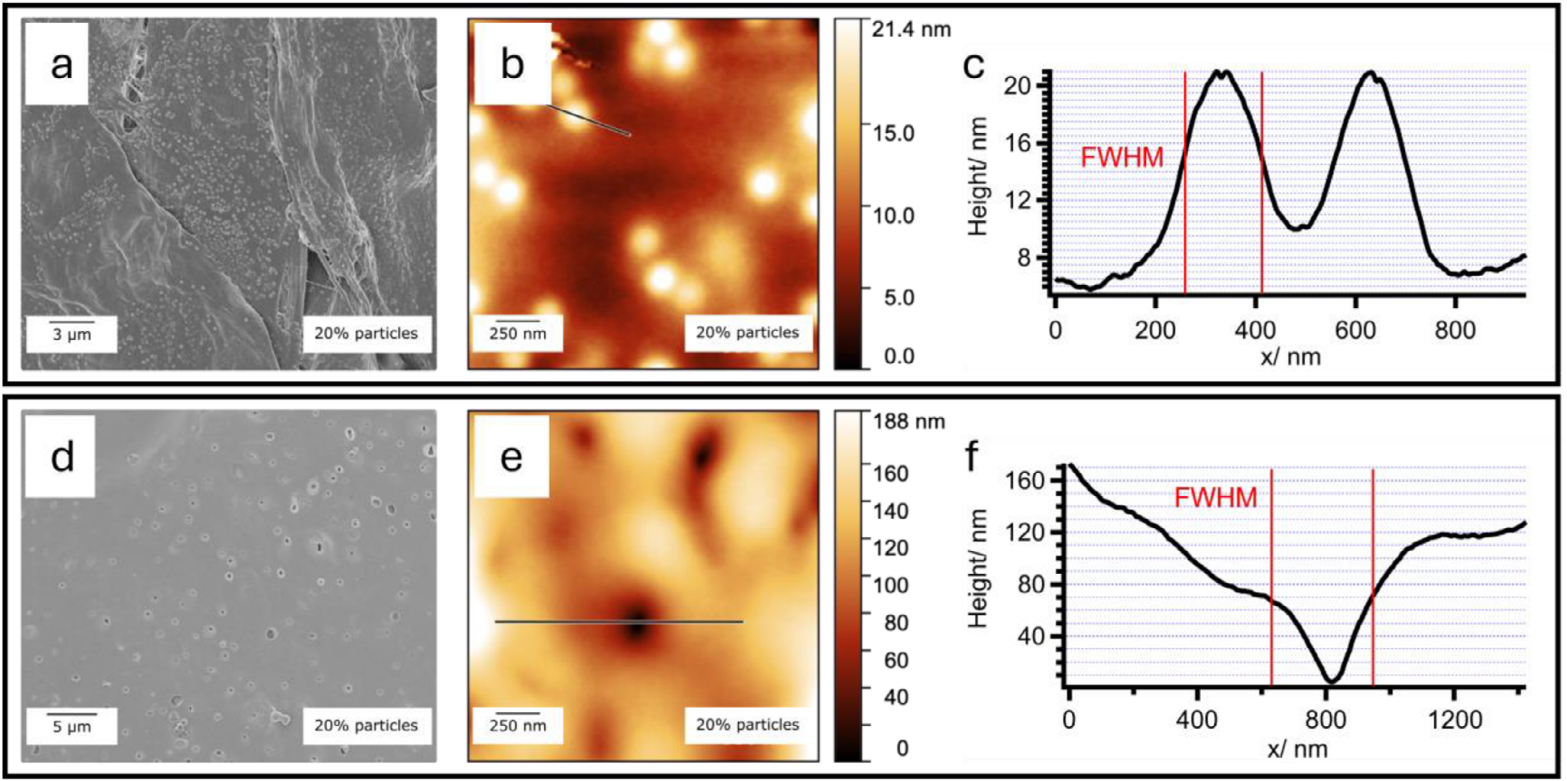
Analysis of the cellulose surface, coated with 20 mass% CSPs with an outer shell of P(*n*BuA‐*co*‐GlyMA). From left to right, the images show scanning electron micrographs (a,d) and AFM scans (b,e), including representative line profiles (c,f) of the coated sample, (a–c) before and (d–f) after etching.

Equivalently, the GlyMA‐containing particles were used to prepare porous coatings onto the cellulose support. To improve the cross‐linking process of the particles after coating, an appropriate cross‐linking system for the GlyMA‐containing polymer was established, using vinyl methacrylate (VMA), a monomer containing two reactive double bonds with different reactivities [36], together with HMPP. In order to obtain a cross‐linked matrix, the cellulose sample, coated with 20 mass% of CSPs, was treated with UV irradiation. Preussmann test of the coated cellulose film revealed the still existing presence of epoxide groups, as indicated by a color change from white to purple (Figure S2). The P(*n*BuA‐*co*‐GlyMA) particles were well distributed on the cellulose fibers, as revealed by SEM and AFM (Figure 3a,b). Here, larger distances between the CSPs were present compared to the previously performed coatings. After etching in 40% HF, a porous coating was successfully formed, with larger pore spacing (Figure 3d,e). The line profiles of the coatings indicated CSP diameters of 108±28 nm (*n* = 10 values). Etching the particles resulted in larger pores with a diameter of 268±152 nm (*n* = 5 values) rather than reducing them to the size of the former core particles. Generally, the polymer matrix swells during the etching process in the aqueous media. The extent of the swelling behavior depends on the matrix, the functional groups of the shell polymers, the thickness and cross‐linking of the particles’ shell, and the cross‐linking density between the CSPs. Therefore, the resulting porous structure of the polymer matrix also depends on the HF etching procedure and the matrix's properties, such as polarity, swelling capacity, and crosslinking density, among others. After the silica cores are etched and the matrix is swollen in the aqueous medium, the hybrid film contains pores, stabilized by the porous matrix structure. Depending on the matrix composition, the pore structure remains intact, collapses, or changes in size during cleaning and drying. However, final pore sizes cannot be directly compared once more parameters were varied.

Equivalent to the previously described coatings, the contact angles of these new coatings were analyzed. The water contact angle of the cellulose paper sheet rose to 57±5° after being coated with the GlyMA‐containing CSPs, thereby showing a more hydrophilic behavior compared to the coatings with CSPs containing HEMA. After etching the cores, the porous coating exhibited a contact angle of 98±3°, comparable to previously investigated coatings using HEMA‐CSPs.

In conclusion, both batches of CSPs, with HEMA or GlyMA in the outer layer, respectively, resulted in coated cellulose films. After etching the silica cores in HF, the water contact angle of the samples increased significantly up to 117° and 98°, respectively, while pores formed on the coating's surface.

## 4 Conclusions

In summary, this work describes a method for designing cellulose‐based materials with a hierarchical porous structure. Therefore, monodisperse core–shell particles were successfully synthesized, using silica spheres as the core material and a soft copolymer shell containing 2‑hydroxyethyl methacrylate or glycidyl methacrylate as two different functionalities of the particle's surface. Coating and subsequent etching yielded porous coatings, as observed with SEM and AFM, with enhanced hydrophobicity. Further improvements in the pore ordering should be achieved, e.g., by using a more controlled coating procedure, so that the CSPs arrange into a close‐packed structure even at higher particle concentrations, as in vertical deposition methods or spin‐coating procedures. Besides its application as a highly porous filtration system, the described preparation method for coated cellulose could also be used for further modifications. For safety reasons, also less hazardous etching conditions based on fluoric agents could be used, such as ammonium bifluoride [37, 38]. The highlights in the described method are the readily addressable functional groups of HEMA and GlyMA, offering routes for post‐functionalization of the coated surface. For this reason, this work paves the way for functional cellulose‐based microfluidic devices and filtration systems with tailored porosity and specific surface chemical properties, paper‐based sensors, and adsorbers.

## Author Contributions

Regina Leiner performed conceptualization (lead); data curation (lead); formal analysis (lead); investigation (lead); methodology (lead); validation (lead); visualization (lead); writing – original draft (lead); writing – review and editing (lead). Derya Kurt performed conceptualization (equal); data curation (equal); formal analysis (equal); investigation (equal); validation (equal); writing – review and editing (supporting). Sebastian Heinz performed data curation (supporting); formal analysis (supporting); methodology (equal); validation (supporting); writing – review and editing (supporting). Volker Presser performed data curation (supporting); formal analysis (supporting); methodology (equal); validation (supporting); writing – review and editing (supporting). Bizan N. Balzer performed data curation (equal); formal analysis (equal); investigation (equal); methodology (equal); validation (equal); visualization (equal); writing – review and editing (supporting). Markus Gallei performed conceptualization (lead); data curation (equal); funding acquisition (lead); investigation (equal); project administration (lead); resources (lead); software (lead); supervision (lead); validation (lead); visualization (equal); writing – original draft (lead); writing – review and editing (lead).

## Conflicts of Interest

The authors declare no conflicts of interest.

## Supporting information




**Supporting File**: marc70293‐sup‐0001‐SuppMat.docx.

## Data Availability

The data that support the findings of this study are available from the corresponding author upon reasonable request.
