# Supplementary material for: Hierarchically Porous Coatings for Cellulose Fibers by Core–Shell Particle Templating
Source: Macromol Rapid Commun. 2026 Apr 27;47(13):e70293. doi: 10.1002/marc.70293 (PMC13331543; doi:10.1002/marc.70293)
Supplement: Supplementary file 1 — Supporting File: marc70293‐sup‐0001‐SuppMat.docx. [file MARC-47-e70293-s001.docx]

Supporting Information

Hierarchically Porous Coatings for Cellulose Fibers

by Core-Shell Particle Templating

Regina Leiner, Derya Kurt, Sebastian Heinz, Volker Presser,

Bizan N. Balzer*, and Markus Gallei*

R. Leiner, D. Kurt, S. Heinz, M. Gallei

*Polymer Chemistry, Saarland University, Campus C4.2, 66123 Saarbrücken, Germany*

E-mail: [markus.gallei@uni-saarland.de](mailto:markus.gallei@uni-saarland.de)

V. Presser

*INM - Institute for New Materials, Saarland University, Campus D2.2, 66123 Saarbrücken, Germany*

*Department of Materials Science and Engineering, Saarland University, Campus D2.2, 66123 Saarbrücken, Germany*

V. Presser, M. Gallei

*saarene, Saarland Center for Energy Materials and Sustainability, Campus C4.2, 66123 Saarbrücken*

B. N. Balzer

*Institute of Physical Chemistry, University of Freiburg, Albertstr. 21, 79104 Freiburg, Germany*

*Freiburg Materials Research Center (FMF), University of Freiburg, Stefan-Meier-Str. 21, 79104 Freiburg, Germany*

E-mail: [bizan.balzer@physchem.uni-freiburg.de](mailto:bizan.balzer@physchem.uni-freiburg.de)

Particle and Coating Analysis


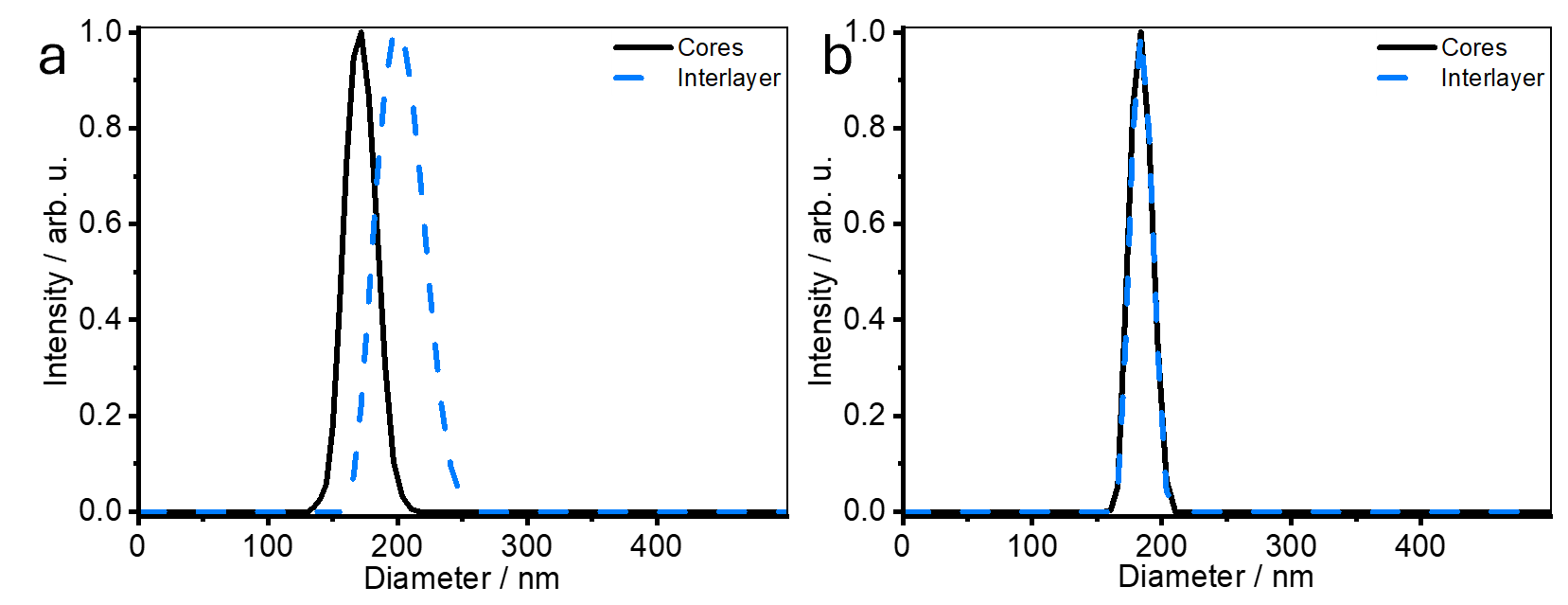


**Figure S1.** DLS analysis of the silica cores and the particles containing an interlayer for the preparation of a) P(EA-*co*-HEMA), and b) P(*n*BuA-*co*-GlyMA) particles.

Preussmann tests

Preussmann tests were performed to demonstrate the presence of the epoxide groups in GlyMA due to the color change to purple after adding 4-(4-nitrobenzyl)-pyridine to the particles:


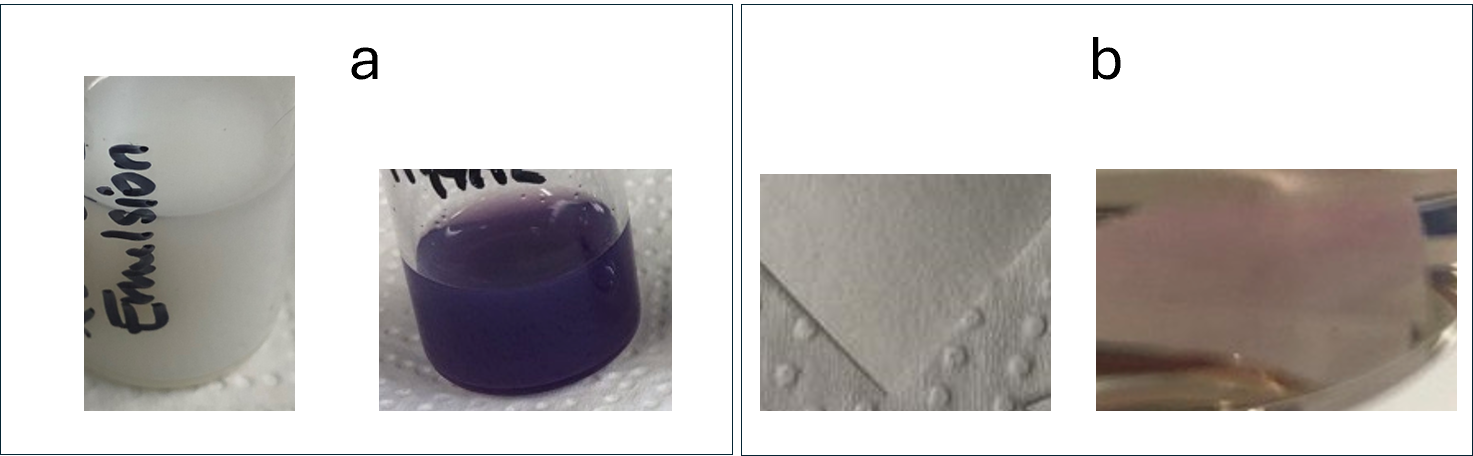


**Figure S2.** Photographs of Preussmann tests (before/after) for proving the presence of epoxide groups using a) the emulsion and b) the coated filter paper.

**Analysis of the coatings**


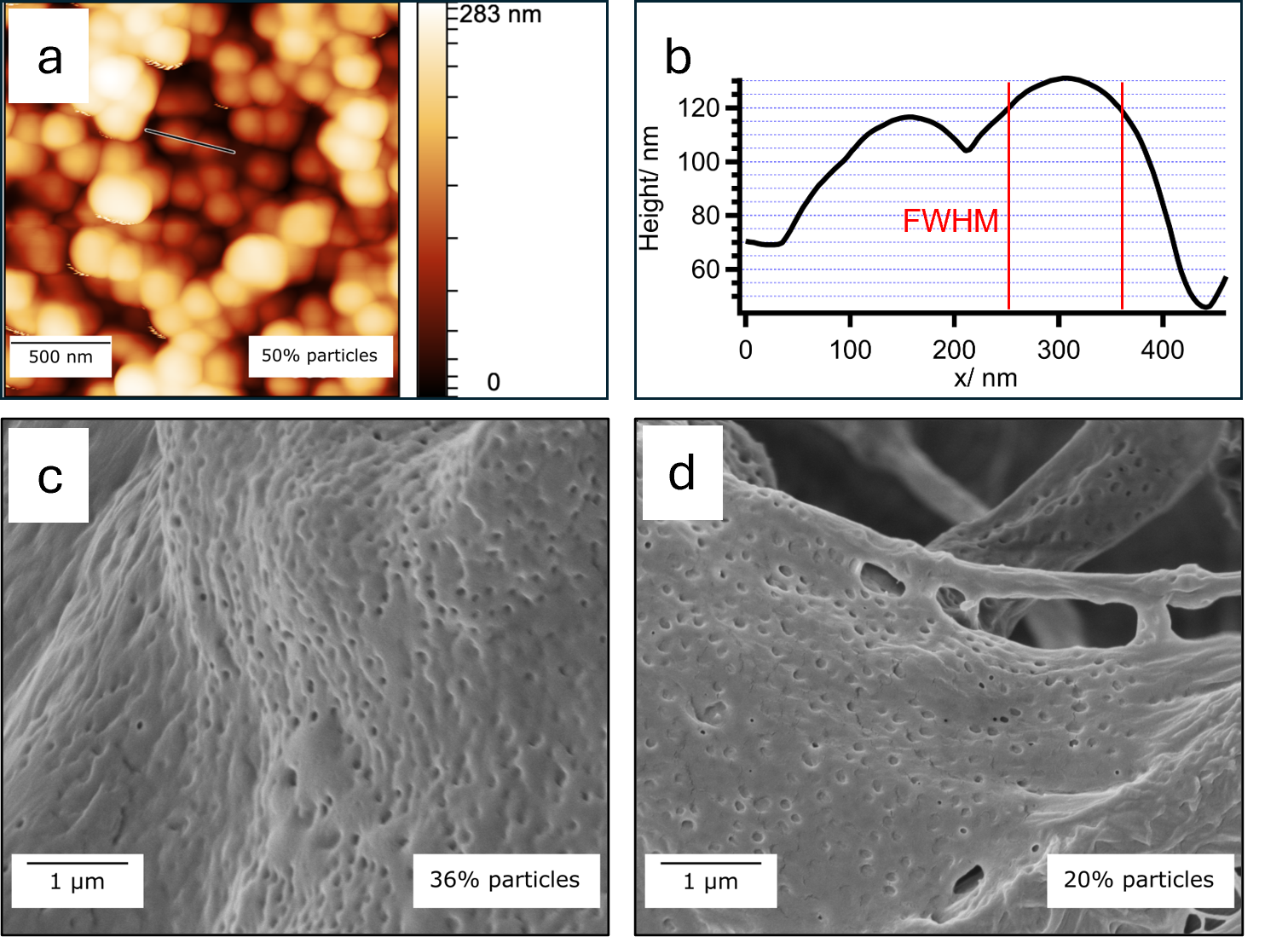


**Figure S3.** Analysis of the cellulose surface, coated with CSPs with an outer shell containing HEMA. a,b) AFM analysis of the coating with 50 mass% of CSPs, including a representative line profile. Scanning electron micrographs of the porous sample after etching using c) 36 mass% CSPs, and d) 20 mass% CSPs.

HF etching of a comparable opal film

Figure S4 shows the analysis of an opal film made of CSPs with silica as the core material and P(EA-*co*-HEMA) as the shell material. The particle emulsion was dried, the resulting particles were mixed with the cross-linking system, extruded, and the extruded filament was pressed in a hot press before cross-linking. After etching the opal film in HF, a porous surface structure formed, whereas the film’s cross-section remained nonporous. Thermogravimetric analysis (TGA) of the film in a synthetic air atmosphere depicted that the residual mass at 590 °C, corresponding to the amount of silica in the film, decreased significantly after the etching process. Still, a significant amount of 18 mass% remained after HF treatment. To conclude, the porous structure within the opal film could have collapsed. This would be the effect of an incomplete cross-linking of the matrix. Another important explanation is that the HF reached only the surface of the films, whereas the silica particles in the cross-section remained.


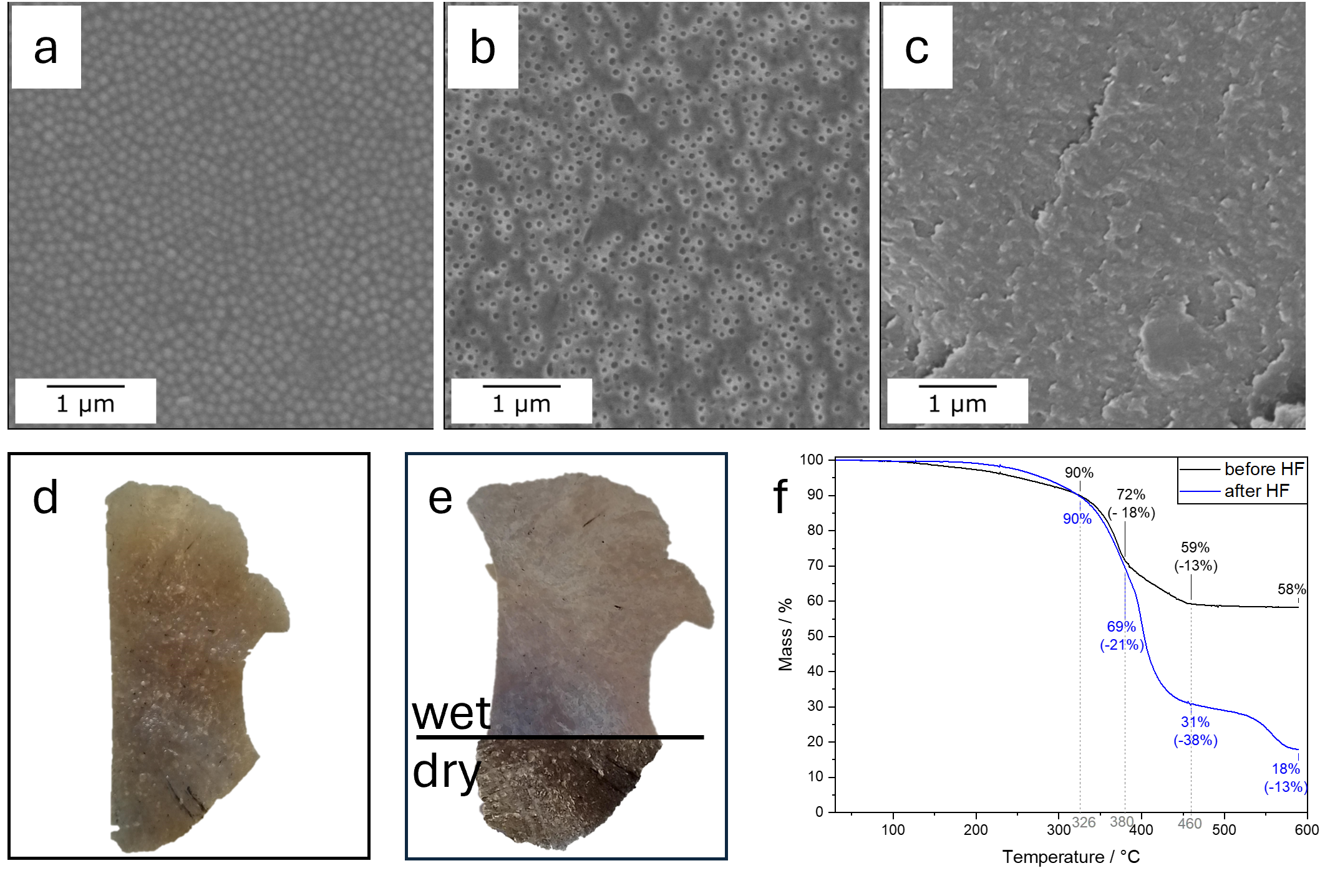


**Figure S4.** Opal Films of P(EA-*co*-HEMA) CSPs with silica cores. a) Scanning electron micrograph of the surface before and b) after HF etching. c) Scanning electron micrograph of the cross-section after etching. d) Photograph of the film before and e) after etching. f) TGA of the film before and after etching.
